# Editorial: Natural products and intestinal mucosal immunity

**DOI:** 10.3389/fimmu.2025.1682978

**Published:** 2025-09-29

**Authors:** Haiqiang Yao, Shanlan Mo, Pinpin Sui, Chunping Wan, Jin-Yi Wan

**Affiliations:** ^1^ School of Traditional Chinese Medicine, Beijing University of Chinese Medicine, Beijing, China; ^2^ National Institute of Traditional Chinese Medicine Constitution and Preventive Medicine, Beijing University of Chinese Medicine, Beijing, China; ^3^ State Key Laboratory of Quantitative Synthetic Biology, Shenzhen Institute of Synthetic Biology, Shenzhen Institutes of Advanced Technology, Chinese Academy of Sciences, Shenzhen, China; ^4^ The University of Texas Health Science Center at San Antonio, San Antonio, TX, United States; ^5^ First Affiliated Hospital of Yunnan University of Traditional Chinese Medicine, Kunming, China

**Keywords:** natural products, intestinal mucosal immunity, intestinal diseases, gut microbiome, intestinal inflammation, gut barrier

The gastrointestinal tract is the largest immunological interface of the body. Mucosal immunity maintains a delicate equilibrium in this surveilled environment by balancing tolerance to commensal bacteria with defense against pathogens (1). Disruption of this equilibrium can trigger the pathogenesis of inflammatory bowel disease (IBD), metabolic disease, and extra-intestinal inflammatory disorders (2). Natural agents, including phytochemicals, herbal extracts, and dietary constituents, are emerging as potent modifiers of intestinal immunity (3). Their pleiotropic mechanisms, favorable safety profiles, and ability to synergize with the host physiology confer specific advantages compared to conventional immunomodulatory approaches. This Research Topic compiles pioneering research that elucidates how natural molecules modulate intestinal immunity at the molecular, cellular, and multi-omic levels. The ten articles included here explore diverse mechanisms from mitochondrial homeostasis and cell death mechanisms to microbiome crosstalk and clinical translation, collectively advancing natural products from empirical remedies to rationally based therapeutics.

At the forefront of intestinal defense, disruption of the epithelial barrier initiates inflammatory cascades. Cai et al. demonstrated that hyperglycemia exacerbates barrier damage by activating neutrophil extracellular traps (NETs). These are extracellular chromatin structures that disrupt tight junctions. The flavonoid baicalin can prevent NET formation by inhibiting histone citrullination, a key modulator of NETosis; this preserves intestinal integrity in diabetes models. Zhou et al. revealed that aberrant mitochondrial dynamics play a critical role in ulcerative colitis (UC) pathogenesis. The accumulation of dysfunctional mitochondria was found to generate reactive oxygen species (ROS), which trigger the NLRP3 inflammasome and perpetuate inflammation. Phytochemicals such as curcumin and resveratrol enhance mitophagy via the PINK1/Parkin and AMPK pathways. This selective mitochondrial elimination reduces epithelial apoptosis and restores redox balance; thus, mitochondrial quality control was established as a target for treating IBD with natural compounds.

The Janus kinase/signal transducer and activator of transcription (JAK/STAT) pathway is a key target of natural compounds that modulate mucosal immunity. According to Long et al., berberine suppresses STAT1/STAT3 phosphorylation, and *Tetrastigma hemsleyanum* polysaccharide induces SOCS1 expression, serving as a natural JAK inhibitor. These agents promote Th17/Treg cell balance and improve colitis without the hematologic toxicity associated with synthetic JAK inhibitors. Pan et al. observed that the flavonoid kurarinone inhibits Th17 differentiation and increases IL-10-producing regulatory T cells by upregulating Blimp-1. Cell death pathways also revealed their multi-mechanistic roles, and Zhao and Lin found PANoptosis, an integrated cell death program involving pyroptosis, apoptosis, and necroptosis, to be a primary force in IBD. The authors demonstrated how berberine blocks caspase-8-mediated epithelial apoptosis, suggesting that natural products may intercept PANoptosis at multiple levels to ensure mucosal viability.

The microbiome, serving as both a target and a mediator of natural products, is essential in regulating intestinal immunity. According to Zhu et al., commensal fungi utilize pattern recognition receptors such as Dectin-1 and TLRs to modulate host gut immunity. Pan et al. demonstrated that kurarinone augments beneficial gut genera such as *Lactobacillus* and *Ruminiclostridium*, contributing to the modulation of intestinal mucosal inflammation. Guan et al. investigated the microbial-metabolite-immune axis of gastroesophageal reflux disease (GERD). Lower GI microbiome dysbiosis may indirectly induce GERD by changing gut dynamics, and this could be mitigated by ingredients in Chinese herbal medicine. These compounds restore microbial richness and enhance mucin secretion and thus illustrate how prebiotic-like natural compounds enhance mucosal protection by modulating the gut microbiome.

Natural compounds demonstrate clinical relevance beyond IBD to iatrogenic diseases such as immune checkpoint inhibitor-induced colitis (irColitis). Dong et al. described how traditional Chinese medicine formulas such as Gegen Qinlian decoction alleviate irColitis by inhibiting JAK/STAT and NF-κB signaling pathways. Similarly, Wang et al. demonstrated that cimifugin acts on the gut-organ axis to modulate UC-associated lung injury by inhibiting JAK1/STAT1-dependent macrophage M1 polarization, underscoring the systemic impacts of gut-targeted immunomodulation with botanical compounds.

Future research priorities for this field encompass several key areas. First is the development of precision-targeted nanoparticle formulations to achieve maximum bioavailability and tissue-selective targeting. Second is engineering protective microbiomes through the synergistic combination of phytochemical prebiotics, probiotics, and other applicable approaches. Third is identifying additional natural products with the ability to modulate intestinal mucosal immunity and establishing a multidimensional methodological approach to elucidate the mechanisms of action, thereby laying a foundation for further clinical applications. Fourth is conducting rigorous clinical benchmarking via randomized controlled trials comparing natural products with biologics in UC, irColitis, and related conditions, to further validate the clinical effectiveness of natural products.

This Research Topic illuminates the cutting-edge advances in this field. Natural products are evolving from complementary therapies to emerge as potent immunomodulatory agents with selective molecular targets. With applications ranging from the inhibition of NETosis and the regulation of PANoptosis to the activation of mitochondria and the alteration of the microbiome, natural products modulate the intestinal immune system with remarkable breadth. The included studies integrate ethnopharmacology with molecular medicine, offering promising approaches to addressing conditions that are associated with gut mucosal immunity.
